# Misinnervation in third nerve palsy: Vertical synergistic divergence

**DOI:** 10.4103/0301-4738.57150

**Published:** 2009

**Authors:** Jitendra Jethani

**Affiliations:** Pediatric Ophthalmology and Strabismus, Dr. Thakorbhai V Patel Eye Institute, Vadodara, India.

Dear Editor,

Congenital third nerve palsies are rare, compared with acquired palsies. When congenital third nerve palsy occurs from injury at birth, there may be rapid, complete recovery, but in many instances, there is incomplete recovery with some evidence of aberrant regeneration, or both.[1]

**Figure 1:**
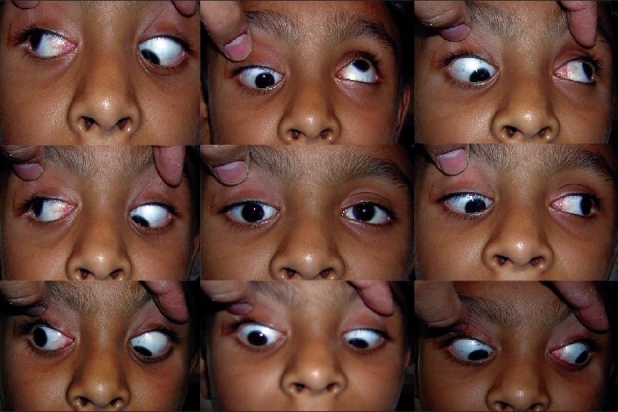
The various movements of eyes in different gazes. Note the large overacting superior obliques in both the eyes

**Figure 2:**
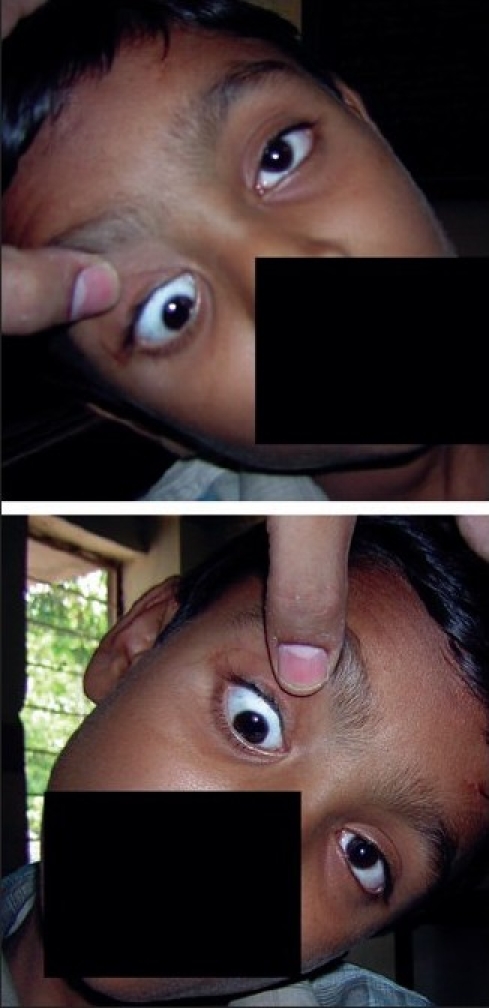
The right hypotropia on head tilt to right and head tilt to left

A 10-year-old child presented with complaints of drooping of upper lid since birth with squint. The child had a best corrected visual acuity of 20/60 in right eye (RE) and 20/20 in left eye (LE). Both the eyes were anatomically normal with normal fundus and no significant ocular torsion. The pupils in both the eyes were normal in size and reacting to light. On ocular motility examination, patient had right hypotropia in primary position with ptosis. On levoversion, he had large left hypertropia with marked superior oblique overaction in RE; underaction of inferior oblique in ductions was noted. On dextroversion, patient had right hypertropia with LE showing superior oblique overaction, a limitation of RE depression [Fig. 1]. Patient showed both eyes superior oblique overaction in dextro and levo depression. The elevation which was restricted in RE was apparently normal in dextroversion. Patient had moderate ptosis (MRD_1_ = 2.5 mm) in RE with poor levator function (4 mm) and frontalis overaction. A lid retraction was noted in downgaze. Also, an A pattern was noted. However, on attempted elevation of LE, there was a downshoot of RE [Fig. 1]. The head tilt test was done but did not reveal any significant change [Fig. 2]. Patient was advised surgery but was lost to follow-up. A forced duction test could not be performed because the child was uncooperative.

Aberrant regeneration is found in congenital third nerve palsies.[1] Wilcox *et al.*,[2] performed electromyography in a boy with congenital adduction palsy and synergistic divergence. They concluded that the bizarre movements were secondary to a misinnervation by a branch of the third nerve with nil or minimal nerve supply by the sixth nerve. Cases with vertical retraction syndrome have also been reported where there is retraction during attempted depression.

The most common acquired eye muscle misdirection syndrome involves the structures innervated by a single cranial nerve, the oculomotor nerve, and is manifested by abnormal patterns of pupillary, lid, and ocular movements.[3]

An important alternative to the misdirection hypothesis involves ephaptic transmission or side-to-side interaxonal cross stimulation. However, ephaptic transmission has not been demonstrated in the third nerve.[3] A third theory states that peripheral nerve injury induces retrograde changes that result in central nervous system (CNS) reorganization.[3] This central reorganization presumably produces synkinesis by unmasking previously encoded connections in the brainstem or higher control center areas.

Currently, congenital cranial dysinnervation disorders (CCDDs) is the term given to such disorders.[4] The CCDDs result from aberrant innervation of the ocular and facial musculature. They generally arise from abnormal development of individual or multiple cranial nerve nuclei or their axonal connections. These disorders appear to result from mutations in genes that are essential to the normal development and/or connectivity of cranial motor neurons.[4]

Our case shows a bilateral superior oblique overaction secondary to third nerve paresis. The downshoot can be explained by the aberrant regeneration of the third nerve with paresis of superior rectus. This prevents any retraction in attempted downgaze. Our case therefore represents a case of vertical synergistic divergence; a variant of vertical Duane's retraction syndrome or similar to horizontal synergistic divergence with no retraction.
